# SCD1, autophagy and cancer: implications for therapy

**DOI:** 10.1186/s13046-021-02067-6

**Published:** 2021-08-24

**Authors:** Francesca Ascenzi, Claudia De Vitis, Marcello Maugeri-Saccà, Christian Napoli, Gennaro Ciliberto, Rita Mancini

**Affiliations:** 1 Department of Clinical and Molecular Medicine, Sant’Andrea Hospital, “Sapienza” University of Rome, 00161 Rome, Italy; 2 Division of Medical Oncology 2, IRCCS “Regina Elena” National Cancer Institute, 00144 Rome, Italy; 3 Department of Medical Surgical Sciences and Translational Medicine, “Sapienza” University of Rome, 00189 Rome, Italy; 4 Scientific Direction, IRCCS “Regina Elena” National Cancer Institute, 00144 Rome, Italy

**Keywords:** Autophagy, Lipid metabolism, cancer

## Abstract

**Background:**

Autophagy is an intracellular degradation system that removes unnecessary or dysfunctional components and recycles them for other cellular functions. Over the years, a mutual regulation between lipid metabolism and autophagy has been uncovered.

**Methods:**

This is a narrative review discussing the connection between SCD1 and the autophagic process, along with the modality through which this crosstalk can be exploited for therapeutic purposes.

**Results:**

Fatty acids, depending on the species, can have either activating or inhibitory roles on autophagy. In turn, autophagy regulates the mobilization of fat from cellular deposits, such as lipid droplets, and removes unnecessary lipids to prevent cellular lipotoxicity**.** This review describes the regulation of autophagy by lipid metabolism in cancer cells, focusing on the role of stearoyl-CoA desaturase 1 (SCD1), the key enzyme involved in the synthesis of monounsaturated fatty acids. SCD1 plays an important role in cancer, promoting cell proliferation and metastasis. The role of autophagy in cancer is more complex since it can act either by protecting against the onset of cancer or by promoting tumor growth. Mounting evidence indicates that autophagy and lipid metabolism are tightly interconnected.

**Conclusion:**

Here, we discuss controversial findings of SCD1 as an autophagy inducer or inhibitor in cancer, highlighting how these activities may result in cancer promotion or inhibition depending upon the degree of cancer heterogeneity and plasticity.

## Background

Autophagy is a catabolic process by which cellular components, including lipids, proteins and organelles, are degraded inside lysosomes and then recycled, contributing to cellular homeostasis [1]. Therefore, autophagy is an essential function for the quality control of cells, but it also has a crucial role in response to nutrient and oxygen deprivation. The degraded and recycled metabolites can provide energy supplies and basic nutrients for cell survival and growth [1]. Nutrient depletion leads to mobilization of free fatty acids (FFAs) from cellular lipid stores to supply energy, thus rendering lipid metabolism and autophagy functionally intertwined processes [2].

Several studies have demonstrated a dual effect of lipids on autophagy. In different tissues and/or cell types (including muscle, pancreas, liver, colon, mammary epithelial cells and neurons) autophagy is upregulated in response to increased FFAs [3–7] while it is downregulated in the presence of high concentrations of specific lipid species. In particular, unsaturated FFAs, such as oleic acid, showed a striking stimulatory effect on autophagy in many cells, at least up to some concentrations (500 μM) [8–10]. Conversely, saturated FFAs (e.g., palmitic acid) remaining in the cytosol at higher concentrations, probably because they were not efficiently incorporated in lipid droplets, suppress autophagy [10]. One of the key regulators of the fatty acid composition of cellular lipids is stearoyl-CoA desaturase 1 (SCD1), also known as fatty acyl-CoA delta-9 desaturase, an endoplasmic reticulum-resident enzyme involved in the synthesis of monounsaturated fatty acids (MUFA) from their saturated fatty acid (SFA) precursors [11].

It has also been observed that autophagy regulates lipid metabolism. Lipophagy, a type of autophagy with a complex role in cell homeostasis, contributes to both the mobilization of stored lipid content and to the translocation of lipids for lysosomal degradation, which prevents excess lipid deposits [12]. Indeed, the inhibition of ULK1 (Unc-51 Like Autophagy Activating Kinase 1), a kinase involved in the initial stages of autophagy, decreases the transcription of SCD1 in liver cells exposed to a lipotoxic environment (e.g., by administration of palmitate), inducing an increased SFA/MUFA ratio and lipotoxic cell death [13]. Lipophagy also provides the fatty acids necessary to support mitochondrial respiration, essential for the differentiation of neutrophils, thus playing a potential role in the treatment of granulocytic leukemia [14].

Given the emerging connection between lipid metabolism and autophagy, and taking into account the dominant role of SCD1 in the cellular lipidic balance, we herein discuss the connection between SCD1 and the autophagic process, along with the modality through which this crosstalk can be therapeutically exploited.

## Role of autophagy in cancer

**Fig. 1:**
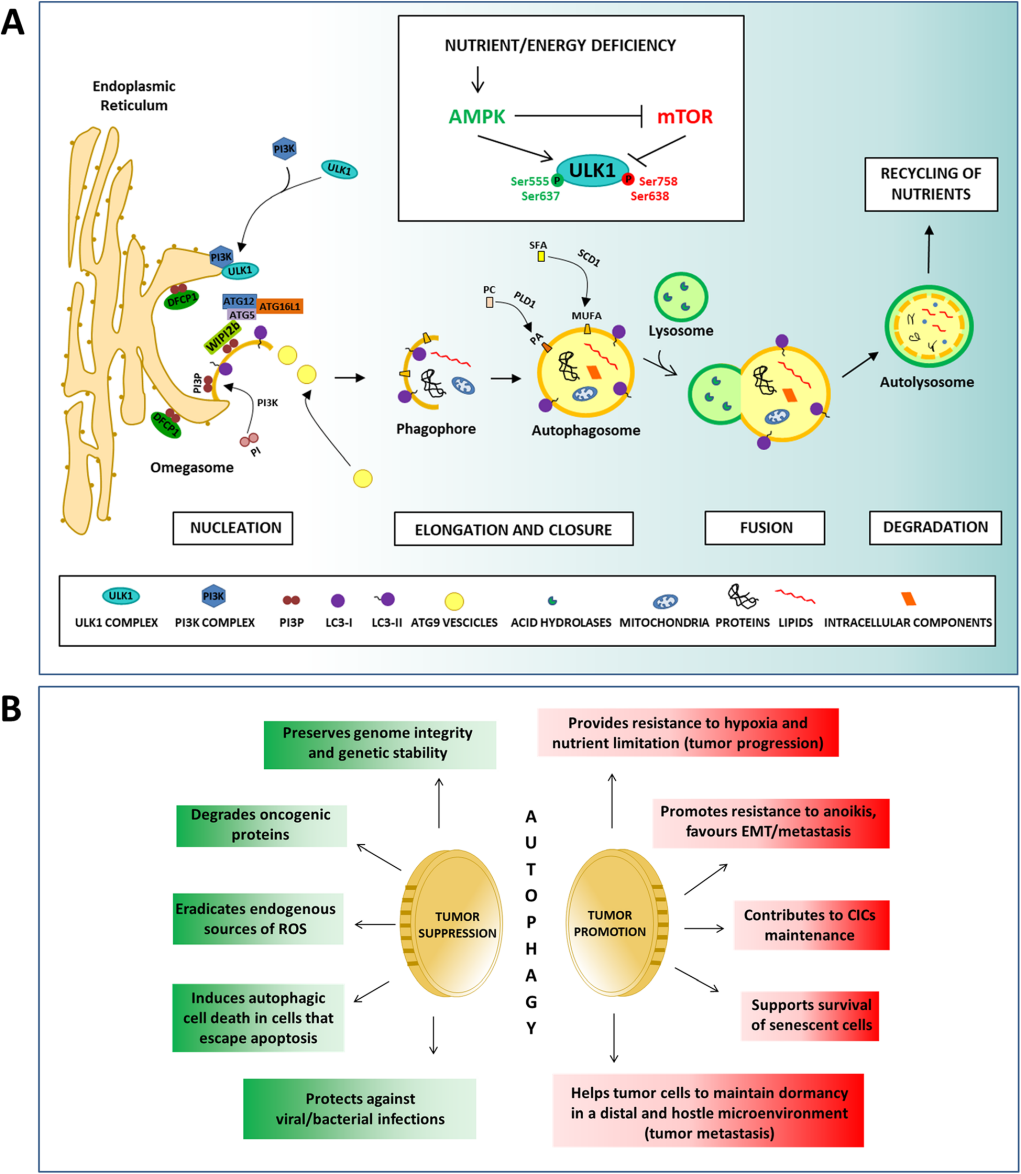
Autophagy **A** The basic autophagy machinery. Autophagy induction is controlled by AMPK and mTOR signaling pathways. Under nutrient/energy deficiency conditions, AMPK indirectly inhibits mTOR and directly activates ULK1 protein by the phosphorylation of activation sites at Ser-555 and Ser-637. Furthermore, ULK1 is a direct target of mTOR, whose inactivation prevents the inhibitory phosphorylation on Serine 638 and 758 of ULK1, promoting its further activation. Once activated, the ULK1 kinase complex translocates to the endoplasmic reticulum, followed by the autophagic PI3K complex I. PI3K complex phosphorylates the lipid phosphatidylinositol to generate a pool of PI3P which drives omegasome formation, recruiting other autophagy effectors and producing the active form of LC3B, commonly called LC3-II. In turn, LC3-II enables the docking of specific cargos and adaptor proteins at the phagophore membrane, such as p62, able to recognize cargos destined to be degraded by autophagy. The continuous assembly of the aforementioned complexes and the delivery of distal membrane compartments allow the phagophore to expand, enclosing a portion of the cytosol, and to form the mature autophagosome. Once formed, the autophagosome fuses with a lysosome, triggering the formation of an autolysosome. After degradation of its content by the action of lysosomal hydrolases, the recycled products are released into the cytosol to be reused by the cell. **B** Autophagy in cancer: two sides of the same coin. Autophagy has a complex and dual role in the pathogenesis of cancer, potentially acting either as a suppressor or a promoter of tumor development. Autophagy protects from malignant transformation by safeguarding genomic stability, removing oncogenic proteins, reducing reactive oxygen species, promoting autophagic cell death and inducing the clearance of intracellular pathogens. Likewise, autophagy favours tumor initiation and progression by providing an alternative energy source in the absence of oxygen and nutrients, promoting the resistance to anoikis, causing the maintenance of Cancer Initiating Cells and supporting the survival of senescent cells, especially in distal sites

Autophagy is a highly conserved self-digesting mechanism responsible for the constitutive turnover of damaged macromolecules and organelles. This catabolic process protects organisms against various cues, including infections, cancer, neurodegeneration, aging and cardiovascular disease [15–25]. Autophagy is articulated in several sequential steps, including nucleation, elongation, closure, fusion and degradation (Fig. 1a). Briefly, an expanding membrane structure (phagophore) enwraps portions of the cytoplasm, incorporating unwanted material. The phagophore expands to form the autophagosome, a double-membrane compartment engulfing cytoplasmic targets (proteins, organelles or pathogens). Afterwards, the autophagosome fuses with the lysosome for cargo degradation and the consequent recycling of nutrients.

Although autophagy is a protective mechanism, it leads to cell death when excessively induced [26]. Thus, this process is finely regulated through a number of progressive stages governed by a complex molecular machinery (Fig. 1a). The role of autophagy in cancer has spurred intense debate in recent years. Given its ability to eliminate potentially harmful cellular components, autophagy is considered a mechanism capable of suppressing the onset of cancer. Consistently, key proteins involved in the autophagic process, including Beclin1, UVRAG, Bif-1 and ATG, act as tumor suppressors by promoting apoptosis in cancer cells [18, 27–31]. Nevertheless, autophagy can also sustain tumor growth by providing nutrients and energy [32–34]. In addition, autophagy promotes the growth and survival of cancer cells exposed to stressful conditions and the maintenance of cancer cell stemness [35–37]. Therefore, depending on the evolutionary stages of cancer and the type of tissue, autophagy plays different, and to some extent opposite roles, that need to be fully elucidated in the attempt of developing targeted therapeutic strategies [25].

These functions, illustrated in Fig. 1b, have induced the scientific community to further explore the impact of autophagy on oncogenesis and tumor progression. Particular emphasis has been placed on the connection between autophagy and cancer-initiating cell (CIC) maintenance/self-renewal in several types of tumors [38]. CICs are usually hidden in the hypoxic core of solid tumors, where they enter in a quiescent state and acquire immune evasive properties [39]. Hypoxia, in addition to regulating the tumor microenvironment, induces the expression of markers involved in autophagy, consequently stimulating this pathway and promoting cell survival [40]. In breast cancer, mammospheres enriched in CICs have an increased autophagy flux compared with adherent cells [41], whereas inhibition of ATG7 decreases 3D tumor spheroid structure formation, both in terms of number and size [42]. Furthermore, some transcription factors commonly associated with pluripotency (e.g., Nanog and Sox2) were tied to induction of autophagy, suggesting a reciprocal regulation between the stem cell program and this catabolic process [37]. These observations support the role of autophagy in the self-renewal of CICs, as well as their tumorigenic capability [42].

A further point is the induction of epithelial–mesenchymal transition (EMT) by the tumor microenvironment. EMT is a reversible cellular program by which tumor epithelial cells acquire mesenchymal traits, along with migratory and invasive properties. This process implies the loss of cell-cell adhesion and the lack of adherence to the extracellular matrix, the inhibition of senescence and anoikis (a programmed cell death induced by cell detachment from the extracellular matrix) and the acquisition of immunosuppressive and CIC features [43]. The tumor microenvironment, composed of inflammatory and immune cells, extracellular matrix, soluble factors and characterized by oxygen deficit, has a crucial role in this cellular transition, through the regulation of different signaling pathways. In this context, evidence indicates that the signaling cascades related to EMT are, at least in part, regulated by autophagy at different levels, favoring the survival of disseminated tumor cells [44]. For instance, the activation of the EMT-associated transcription factors Slug and Snail induces the acquisition of a CIC phenotype and activates autophagy. In turn, autophagy regulates EMT both in terms of activation and inhibition [44]. In this perspective, the dual role that autophagy plays in cancer may be rooted in the regulation of this process.

## Lipids and lipid metabolism enzymes in the regulation of the autophagic machinery

The regulation of the autophagic apparatus is mediated, during key phases, by lipids or lipid metabolism enzymes [45]. Lipids are important for the recruitment of effectors to membranes. For instance, the phospholipid PI3P controls the assembly of scaffold proteins on autophagic membranes, favouring the biogenesis of the autophagosome (Fig. 1a). PI3P is mainly synthesized via phosphorylation of phosphatidylinositol (PI) at the 3′ position of the inositol ring by class III phosphoinositide 3-kinase (PI3K). PI3K is often mutated in cancer, triggers signalling cascades that alter tumor metabolism, and has been clinically validated as an important therapeutic target [46]. Small molecules targeting PI3K have been shown to inhibit autophagy [47, 48] and to contribute to cancer cell death [47].

Another important function concerns the covalent modifications to which some proteins are subjected [45]. For example, LC3 (Microtubule-associated protein 1A/1B-light chain 3) is conjugated to the lipid phosphatidylethanolamine (PE), which triggers its stable anchorage to the phagophore membrane (Fig. 1a). This modification allows the phagophore to enlarge, forming a mature autophagosome.

A further mechanism by which lipids control autophagy involves modifications of physiochemical properties of lipid bilayers, including viscosity, rigidity, and thickness [45]. These features are widely determined by the types of lipids found in the membranes. For example, phosphatidic acid (PA), through its “cone” shape, tends to promote negative curvatures of the membranes, facilitating the budding or fusion of vesicles (Fig. 1a). Phospholipase D (PLD1) is the main lipid enzyme responsible of PA production, catalysing the hydrolysis of phosphatidylcholine (PC). PLD1 is considered a positive modulator of autophagy, since its genetic removal is associated with a reduction in the size and number of autophagosomes in the livers of starved mice [49]. Moreover, elevated PLD1 activity and expression have been observed in many tumors, where its inhibition reduced cell proliferation and migration. Consistently, the targeting of both PLD1 and autophagy, synergizing in inducing tumor cell apoptosis and tumor regression, has been proposed as potential anticancer therapy [50]. Similarly, a number of other lipid enzymes have been associated with the regulation of autophagy in cancer, including Stearoyl-CoA desaturase 1, as discussed below in the next sections.

### Stearoyl-CoA desaturase 1

#### Structure and biochemical reaction

**Fig. 2:**
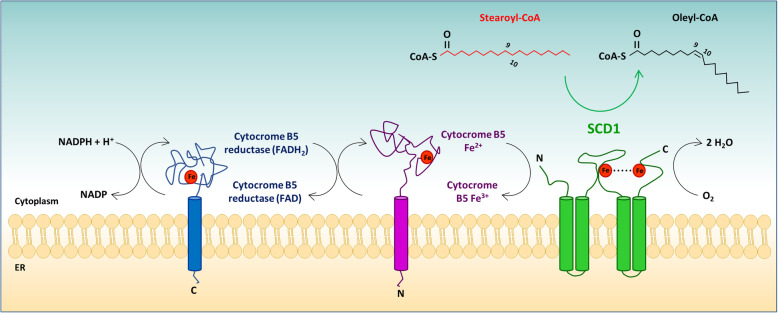
Desaturation of fatty acids by stearoyl CoA desaturase (SCD). SCD1 catalyzes the introduction of a double bond between carbons 9 and 10 of a saturated long chain acyl CoA, such as stearyl CoA. In the reaction, two electrons flow through an electron transport-desaturase complex composed by cytochrome b5 reductase, cytochrome b5 and SCD1. The final acceptor of the electrons is molecular O_2_, which is reduced to H_2_O

SCD, also known as 9-fatty acyl-CoA desaturase, is an iron-containing endoplasmic reticulum-bound enzyme that catalyzes the introduction of a double bond in the cis-9 position of saturated fatty acyl-CoAs [11, 51] (Fig. 2). The mechanism of desaturation involves NADPH, the flavoprotein cytochrome b5 reductase, the electron acceptor cytochrome b5 and molecular oxygen. This reaction is aerobic, as it requires molecular oxygen; however, the latter is not incorporated into the fatty acid chain but is released in the form of water [11]. The desaturation of a fatty acid occurs through a series of redox reactions, during which two electrons flow sequentially from NADPH to the cytochrome b5 reductase (a flavoprotein, FADH_2_), then to the electron acceptor cytochrome b_5_, to SCD, and finally to O_2_, which is reduced to H_2_O. The enzymatic complex first removes a hydrogen atom at the C-9 position and then removes the second hydrogen atom from the C-10 position. The result is the introduction of a double bond at the 9,10 position into a spectrum of methylene-interrupted fatty acyl-CoA substrates [11]. The preferred substrates are palmitoyl- and stearoyl-CoA (palmitate and stearate), which are then converted into palmitoleoyl- and oleoyl-CoA (palmitoleate and oleate), respectively [11].

The SCD protein is localized exclusively in the endoplasmic reticulum, where it is anchored to the membrane through four transmembrane domains [51] (Fig. 2). Both the amino and carboxyl-terminal domains and eight catalytically important histidine residues (hexagonal shapes), which collectively bind iron within the catalytic center of the enzyme, are oriented toward the cytosol. Therefore, the cytosolic domain provides a structural frame for the regioselectivity and stereospecificity of the desaturation reaction [51].

Two SCD isoforms have been identified in human tissues: SCD1 and SCD5 [52–54]. SCD1 is the main isoform, ubiquitously expressed, with a prevalence in adipose tissue, heart, brain, liver and lungs. SCD5 is poorly expressed in adult human tissues and is mostly restricted to the brain and pancreas. While little information is available on the physiological role of SCD5, the biological functions of SCD1 and its involvement in pathological processes are intensively investigated.

#### SCD1: biological function and involvement in cancer

SCD1 promotes the biosynthesis of MUFAs (i.e., palmitoleate and oleate) from their SFA precursors (i.e., palmitate and stearate). MUFAs represent the substrates for the synthesis of various lipids, including phospholipids (PLs), diacylglycerols (DAGs), triacylglycerols (TAGs) and cholesteryl esters (CEs), which represent basic components of biological membranes, as well as a cellular energy source and signaling molecules [55]. Therefore, the activity of SCD1 can influence cellular membrane physiology and signaling, leading to broad effects on human physiology.

SCD1 is a key factor in lipid metabolism and body weight control. High levels of SCD1 are found in the skeletal muscle of obese subjects [56] and correlate with the development of hypertriglyceridemia, atherosclerosis, and diabetes [57]. Accordingly, SCD1-deficient mice showed reduced adiposity, increased insulin sensitivity and resistance to diet-induced obesity [58, 59].

Several studies have shown that SCD1 fuels cancer cell proliferation, tumor growth and metastasis [60–64] (Table 1). Increased expression of SCD1 has been correlated with cancer aggressiveness and poor prognosis across a range of tumors [64–68]. Moreover, SCD1 promotes the maintenance/acquisition of stem-like features, including chemoresistance and self-renewal. In non-small-cell lung cancer, CICs are characterized by SCD1-mediated stabilization and nuclear translocation of YAP/TAZ, and the consequent activation of downstream factors. Conversely, pharmacological inhibition of SCD1 with the small molecule SCD1 inhibitor MF438 induces the degradation of YAP/TAZ [69], promotes the selective apoptosis of ALDH-positive cells [70], and reverts cisplatin resistance [71]. Likewise, *BRAF*-mutated melanoma cell lines growing under 3D conditions and enriched in CICs overexpressed SCD1, exhibited resistance to BRAF and MEK inhibitors [72].

**Table 1:** SCD1 and cancer. Signalling pathways regulated by SCD1 in cancer promotion and development

FUNCTION	EFFECT ON CANCER CELLS	SIGNALLING PATHWAYS INVOLVED	REFERENCES
TUMOR FORMATION	PROLIFERATION	EGFR ERK1/2 MAPK PI3K/AKT CYCLIN D1/CDK4	[60–62, 64]
TUMOR GROWTH	PROLIFERATION	EGFR ERK1/2 MAPK PI3K/AKT CYCLIN D1/CDK4	[61, 62, 65]
TUMOR METASTASIS	MIGRATION AND INVASION	PI3K/AKT GSK3-Β/Β-CATENIN	[60, 63]
TUMOR SUBSISTENCE	MAINTENANCE OF STEM PROPERTIES	WNT/Β-CATENIN/HIPPO NF-ΚB/ALDH1A1	[63, 69–72]

#### Regulation of autophagy by SCD1

The connection between SCD1 and the autophagic process was originally demonstrated in *Drosophila*. Kohler and colleagues observed that knock-out of a *Drosophila* SCD homolog, Desat1, suppressed autophagy, suggesting a role for Desat1 in controlling lipid biosynthesis and/or signaling necessary for autophagic responses [73]. Subsequently, Ogasawara et al. investigated the role of SCD1 in the autophagic process in different mammalian cell lines, including mouse embryonic fibroblasts, NIH3T3 and HeLa cells. Although in SCD1 knocked-down HeLa cells a complete suppression of autophagy was not observed, probably owing to the activity of SCD isozymes (see above) or residual SCD1 activity, the administration of an SCD1 inhibitor in murine fibroblasts strongly inhibited starvation-induced autophagy, resulting in a defective translocation of ULK1 and P62/SQSTM1 to sites of autophagosome formation. Moreover, this effect was reversed by overexpression of SCD1 or supplementation with oleic acid, the catalytic product of SCD1. The activity of SCD1 in autophagy was proposed to be restricted to the early stages of autophagosome formation by i) increasing membrane fluidity and facilitating the autophagosome formation on the endoplasmic reticulum; and ii) generating membrane curvatures through the production of truncated cone-shaped fatty acids, such as oleic acid [74]. The same research group also investigated the autophagic phenotype of the yeast mutant of OLE1, an orthologue of SCD1. They observed the failed recruitment of ATG9 (Autophagy-related protein 9) on the pre-autophagosomal structure, with consequent defects in elongation of the isolation membrane and in autophagosome formation [75]. The implication of SCD1 in this cellular mechanism has also been investigated in pancreatic β-cells, in which autophagy is essential for correct architecture and functioning [76]. SCD1 inhibition affects the autophagic flux at the level of autophagosome-lysosome fusion, enhancing β-cell dysfunction and palmitate-induced apoptosis. The link between SCD1 inhibition and autophagy/apoptosis crosstalk involves changes in intracellular membrane phospholipids and the induction of ER-to-mitochondria stress signaling. In particular, the decrease in the SCD1 activity, in addition to inducing a defective autophagosome-lysosome fusion and impairing autophagy, leads to dysfunctional ER stress, mitochondrial collapse and the activation of intrinsic apoptosis [76].

Ever since, other studies have highlighted the involvement of fatty acids metabolism in the regulation of autophagy [77, 78]. For example, Santano et al. discovered that saturated fatty acids, but not unsaturated fatty acids, can activate a type of non-canonical autophagic response that uses an intact Golgi apparatus and is independent of Beclin-1, both in vitro and in vivo [77]. More recently, it has been shown that the FAA1 enzyme, an acyl-CoA synthetase, accumulates in the nucleated phagophores and induces the activation of the fatty acids necessary for their expansion, thus allowing the initiation of autophagy [78].

## The controversial role of SCD1-mediated autophagy in cancer and future research perspectives

Although several studies have suggested a positive regulation of autophagy by SCD1, this modulation appears to be controversial in cancer. While a mild autophagy reduction was observed following SCD1 inhibition [74] in cervical cancer HeLa cells, an opposite trend was reported in other tumor cell lines.

In the perspective of autophagy as a pro-survival mechanism, Ono et al. found that the inhibition of SCD1 (using both the small molecule T-3764518 and SCD1-KO) in the colon cancer cell line HCT-116 accelerated the autophagic process through the activation of AMPK, thus escaping the cytotoxic effects of SCD1 inhibition [79]. The authors speculated that the excessive accumulation of saturated fatty acids, due to SCD1 inhibition, triggers an AMPK-mediated compensative resistance mechanism able to block further fatty acid synthesis while simultaneously activating autophagy. This led to the mitigation of lipotoxicity and increased cell survival. This study concluded that targeting SCD1 should be optimized by combining inhibitors of the autophagic process. This combination may overcome resistance mechanisms, thereby inducing cell death.

As mentioned above, autophagy may also act as a promoter of cell death [30, 31]. For instance, Huang et al. reported that the pharmacological inhibition of SCD1 with CAY10566 promoted apoptosis of human hepatocellular carcinoma (HCC) cells in an autophagy-dependent manner [80]. In particular, the repression of SCD1 stimulated the autophagic process, promoting an opposite effect compared to what was observed by Ono et al., i.e., the induction of cell death rather than cell survival. These authors have also correlated the increased expression of SCD1 in HCC with a shorter overall survival in patients, suggesting that the autophagy suppression, mediated by SCD1, may contribute to the development and progression of cancer. From this perspective, the inhibition of SCD1 as a clinical treatment should be considered in association with autophagy activators, at least in HCC. A similar observation was also made by Pisanu et al. [71]. In particular, inhibition of SCD1 with MF438 led to activation of the endoplasmic reticulum stress response coupled with a marked increase in autophagy, as indicated by elevated LC3-II levels. Of note, this activation of the autophagy process was associated with the selective apoptosis of CICs [71].

Different explanations may reconcile the discrepancies observed when investigating the relationships between SCD1 and autophagy in cancer. In the next section, we describe some of the regulatory mechanisms that may explain such inconsistencies.

### Different function and tissue distribution of SCD1

**Fig. 3:**
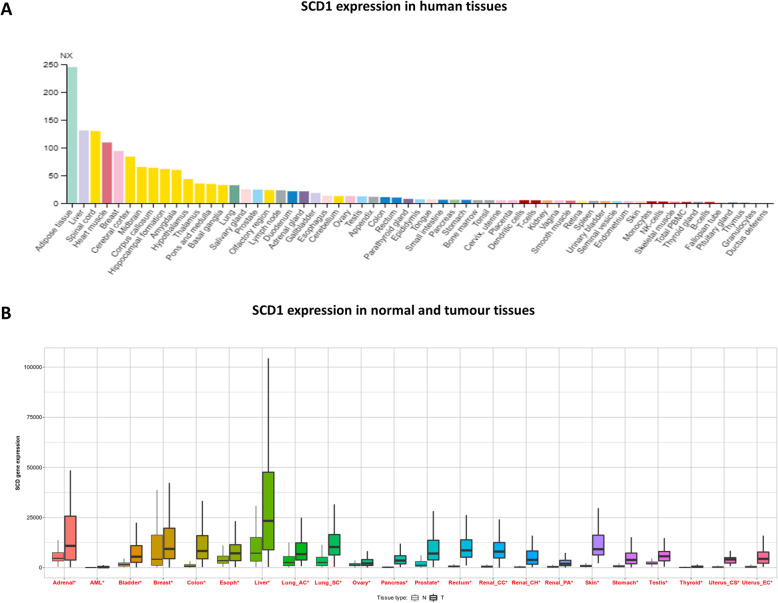
SCD1 expression. **A** Summary of the mRNA expression pattern of SCD1 across the analyzed normal tissues. Consensus Normalized eXpression (NX) levels for 55 tissue types and 6 blood cell types, created by combining the data from the three transcriptomics datasets (HPA, GTEx and FANTOM5) using the internal normalization pipeline. Colour-coding is based on tissue groups, each consisting of tissues with functional features in common [81]. **B** The expression range for SCD1 across tissues in available normal and tumor RNA-Seq data. Significant differences by Mann-Whitney U test are marked with red* [82]. Source: adapted from 81, 82

The variation in SCD1 gene expression levels across tissues reflects different metabolic phenotypes. Although SCD1 is a ubiquitous metabolic enzyme, it plays a key role in lipogenic tissues, such as adipose tissue and liver, where it is expressed at high levels [81] (Fig. 3a). These tissues are highly predisposed to the synthesis of fatty acids, triglycerides and cholesterol. Other districts, such as skeletal muscle, represent important sites for glutaminogenic and carbohydrate metabolism and are consistently characterized by lower levels of SCD1.

**Fig. 4:**
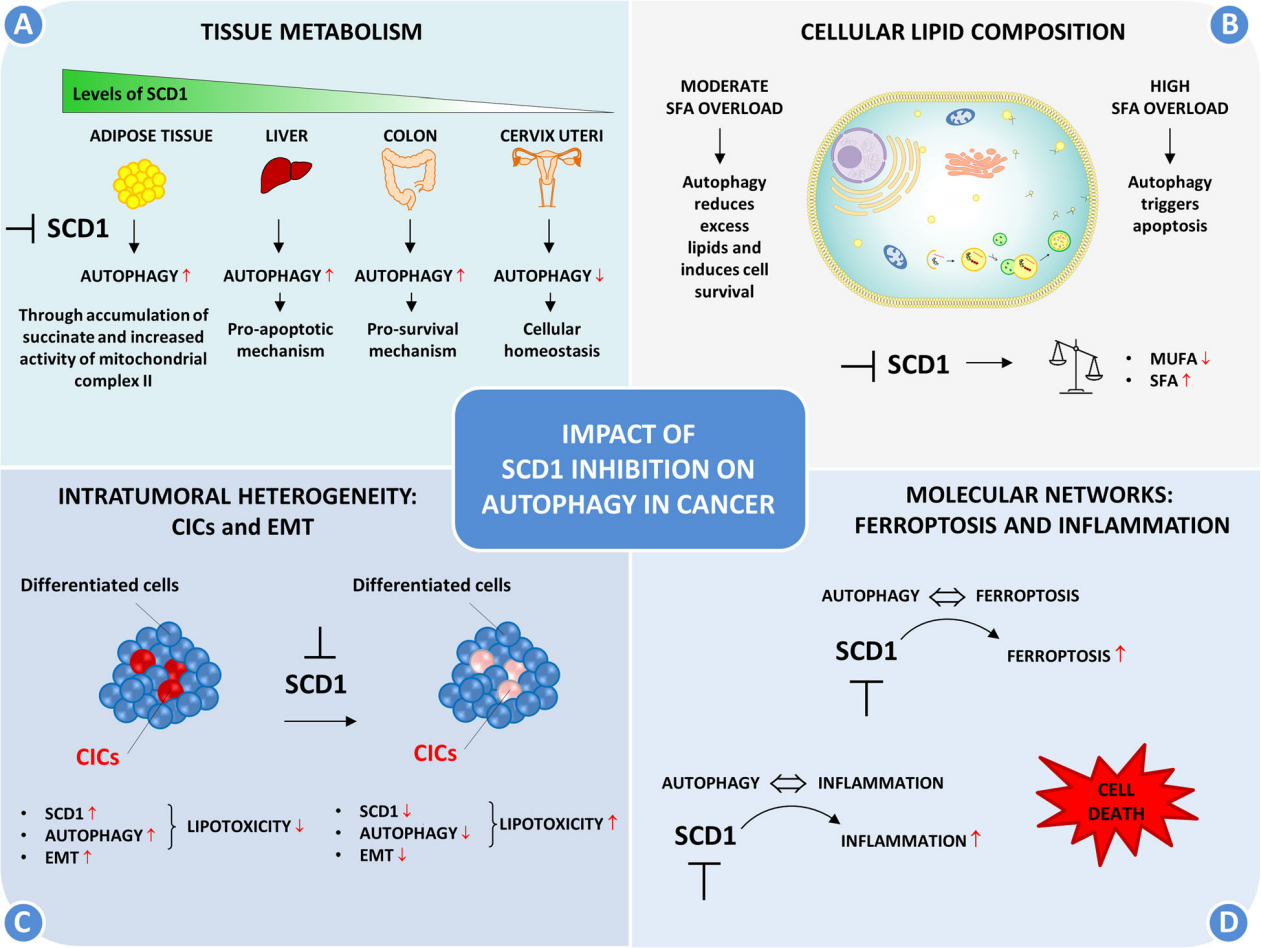
Impact of SCD1 inhibition on autophagy in cancer. Several factors may contribute to autophagy regulation following SCD1 inhibition. **A** Depending on the type of tissue and the differential expression of SCD1, inhibition of SCD1 has different repercussions on autophagy. **B** Cellular lipid content drives cell-fate through regulation of autophagy: survival or cell death. **C** Differential response to SCD1 inhibition is based on the degree of cell differentiation. **D** SCD1 depletion integrates with other cellular pathways, including autophagy, inflammation and ferroptosis

In this scenario, it is plausible that SCD1 function is tissue-dependent and that it plays a different regulation in autophagy depending upon the biological context. A conceivable hypothesis is that tissues expressing high levels of SCD1, such as the liver, are highly dependent on the enzyme. In these contexts, SCD1 inhibition makes them particularly susceptible to autophagy activation for inducing cell death (Fig. 4a). Conversely, in tissues characterized by low SCD1 levels, such as colon and cervix (Fig. 3a) [81], the inhibition of SCD1 may have a limited effect on cell viability. Here, autophagy prevalently participates in cellular homeostasis (Fig. 4a). Furthermore, by comparing normal tissues and their neoplastic counterpart, it has been observed that the expression of SCD1 increases in almost all tumor tissues, even if the largest increase was found in the liver (Fig. 3b) [82], further strengthening the previous hypothesis.

This picture is further complicated by the fact that cancer cells can acquire heterogeneous metabolic preferences and dependencies that markedly differ from the original tissue [83]. Indeed, it is known that mutations in oncogenes and tumor suppressors can stimulate cell-autonomous metabolic reprogramming [83]. In this way, different oncogenic drivers can produce divergent metabolic phenotypes, contributing to metabolic heterogeneity among tumors arising in the same tissue. On the other hand, tumors arising in different tissues may display divergent metabolic features even if they carry the same oncogenic drivers.

### Contribution of CICs and EMT

Tumor heterogeneity may account for inconsistent findings observed in cancer. In this context, the contribution of the CIC compartment deserves increased consideration. As compared to differentiated cells, CICs are characterized by an upregulation of SCD1 coupled with increased autophagic process [38, 70], suggesting that both pathways contribute to their survival by decreasing the degree of lipotoxicity (Fig. 4c). Consequently, the inhibition of SCD1 in a heterogeneous population of tumor cells may produce different effects in the two types of cells. In particular, inhibition of SCD1 in CICs makes them extremely vulnerable to lipotoxicity cell death. Conversely, more differentiated cancer cells exhibit a lower SCD1 dependency, resulting to be less affected and more resistant to abrogation of SCD1 function (Fig. 4c). This implies that the size and the plasticity of the stem cell compartment could be decisive for the effect on the entire cell population following SCD1 inhibition.

EMT may also contribute to tumor plasticity given its reversible nature. EMT is accompanied by significant changes in lipid metabolism. It has been observed that elevated levels of SCD1 promote the migration and invasion of cancer cells [84], while its inhibition with A939572 suppresses this phenomenon in lung cancer [85] (Fig. 4c). Moreover, EMT requires autophagy to sustain the viability of potentially metastatic cancer cells. For instance, a connection between EMT-like phenotype and high autophagy flux has been reported in renal cell carcinoma [44]. Therefore, the balance between the EMT process and its reverse, the mesenchymal–epithelial transition, may be influenced by the depletion of SCD1 and consequently affect the autophagic process.

### Cellular lipid composition and lipotoxicity

Numerous lines of evidence indicate the ability of fatty acids, both saturated and unsaturated, to modulate autophagy [86]. Mice fed with a high-fat diet showed the formation of double-membrane autophagosomes in the liver and increased levels of LC3-II, a marker of autophagosome formation and activity of autophagic flux [10]. Autophagy is believed to be a protective mechanism against lipotoxicity, a condition in which excessive accumulation of lipids occurs in non-adipose cells, leading to cellular dysfunction and death [2, 8]. While saturated fatty acids have been related to adverse health effects, unsaturated fatty acids, especially monounsaturated and ω-3 polyunsaturated, are believed to be protective [87]. Moreover, several sets of evidence reported a different regulation of autophagy by saturated and unsaturated lipids, revealing the former as activators and the latter as inhibitors [8–10].

In the field of cancer, the relationship between metabolism of fatty acids and autophagy remains a controversial issue. For instance, in glioblastoma a chemopreventive and therapeutic role was demonstrated for ω3-polyunsaturated fatty acids, which induce, both in vitro and in vivo, apoptosis of tumor cells through an increased autophagic activity [88]. In a further study, another polyunsaturated fatty acid, docosahexaenoic acid monoglyceride (MAG-DHA), was found to induce both apoptosis and autophagy in breast cancer cells. In this case, however, autophagy acts as a suppressor of apoptosis [89].

On this ground, the observed dual effect regarding the regulation of autophagy by SCD1 in cancer may depend on the lipid composition and the degree of lipotoxicity of the cells, configuring lipids as stress sensors involved in the cell-fate decision (Fig. 4b). Based on this concept, it can be assumed that if the level of SFA overload is moderate, autophagy could intervene by reducing excess lipids and inducing cell survival; conversely, if the level of lipotoxicity is excessive, autophagy could trigger apoptosis. SCD1, by finely regulating the cellular balance between saturated and unsaturated fatty acids, could therefore have a strong directional impact on the functionality of the autophagic compartment (Fig. 4b).

### Regulation of metabolites

The regulation of autophagy by SCD1 can also occur through the modulation of specific metabolites. In the adipose tissue, inhibition of SCD1 caused an accumulation of succinate in the adipocyte progenitors, with consequently increased activity of the mitochondrial complex II and a shift in the differentiation fate from white to brown adipogenesis [90]. The overexpression of SCD1, observed in most tumors, may instead induce a reduction of succinate and a lower activity of complex II. Accordingly, a reduction in the activity of complex II was found in several tumors, such as renal carcinoma and breast cancer [91, 92]. Furthermore, emerging evidence suggests that this biological phenomenon has significant repercussions on autophagic activity (Fig. 4a). Recently, it has been noticed that the inhibition of the mitochondrial complex II through the toxin 3-nitropropionic acid (3-NPA), resulted in incomplete autophagy and lack of neuroprotection [93]. Taken together, these observations suggest that SCD1-modulated metabolites may influence the autophagic process, thus making their interconnection in cancer even more complex and intriguing.

### Ferroptosis

Another biological process that connects SCD1 and autophagy is ferroptosis, a type of iron-dependent programmed cell death characterized by the accumulation of lipid peroxides [94]. Autophagy has recently been shown to play a crucial role in the induction of ferroptosis by regulating cellular iron homeostasis (mostly by inducing ferritin degradation), as well as by controlling reactive oxygen species generation [94]. In turn, prolonged iron-mediated reactive oxygen species generation can induce autophagy, triggering an intense crosstalk between these two processes, which eventually culminates in the induction of cell death [95, 96].

Recent studies have also shown the involvement of SCD1 in the regulation of ferroptosis [97, 98]. Tesfay and colleagues correlated high expression levels of SCD1 found in different ovarian cancer isotypes with the synthesis of lipids implicated in protection from ferroptosis, as a mevalonate metabolite CoQ10. Moreover, they reported that inhibition of SCD1 decreased the mevalonate pathway, inducing both an increase in apoptosis and ferroptosis [97]. These results were corroborated by in vivo ovarian cancer xenograft studies. In particular, the highest therapeutic efficacy was achieved when the SCD1 inhibitor A939572 and the ferroptosis inducer erastin were administered in combination rather than as a single agent [97, 99]. Additionally, Wohlhieter et al. identified SCD1 as an essential gene for survival and ferroptosis protection in STK11/KEAP1 co-mutant lung adenocarcinoma [98], a subset of tumors characterized by resistance to available therapies and early death [100].

These lines of evidence suggest a link between SCD1, autophagy and ferroptosis, where the latter can play a decisive role (Fig. 4d).

### Inflammation

Inflammation is defined as one of the hallmarks of cancer. Many studies have demonstrated that the inflammatory microenvironment induces the initiation of tumors and contributes to their progression and metastatic spread [101]. Likewise, lipid metabolism alterations have emerged as essential for tumor development and evolution. Indeed, obesity and associated metabolic conditions have been shown to increase the risk of cancer, negatively impacting the prognosis of overweight patients [102].

Lipid metabolism and inflammation appear to be closely related. The excess of adipose tissue induces low chronic inflammation, increasing the circulating levels of proinflammatory cytokines, such as IL-6, IL-8, TNF-α, as well as angiocytokines (e.g., factors such as VEGF), which have pro-tumorigenic effects [102]. Regarding SCD1, it has been found that the selective removal of SCD1 in the intestinal epithelium enhanced the vulnerability to inflammation, denoting, in normal conditions, a function of metabolic protection against inflammation and intestinal tumorigenesis for SCD1 and its product oleic acid [103]. Similarly, the cutaneous depletion of SCD1 in SKO mice (skin-specific deficiency of SCD1) induced elevated levels of IL-6 in hair follicle cells and in keratinocytes, which in turn determined increased lipolysis of the white adipose tissue and a decreased whole-body adiposity [104].

Autophagy and inflammation are also strongly interconnected [105]. This is corroborated by the fact that several chronic inflammatory disorders are associated with autophagy dysfunction. The molecular crosstalk between autophagy and inflammation ensures a vigorous immune response, able to modulate both the antitumor innate and adaptive immunity. Autophagy regulates different components of the same inflammatory signaling cascade depending on the biological context, making this process, also from this point of view, complex and somewhat ambiguous [105].

These observations suggest that lipid metabolism, inflammation and autophagy are part of a common molecular circuit, and that the modulation of autophagy by SCD1 is the result of this intricate network (Fig. 4d).

### Future research perspectives

According to the available evidence, tumor heterogeneity and plasticity plausibly play a decisive role in the way in which the tumor mass reacts to the action of SCD1 inhibitors. Underlying the phenomenon of tumor heterogeneity is the concept that different cancer cells show diverse phenotypic profiles, in terms of cellular morphology, gene expression, metabolism, motility, proliferation and metastatic potential. Furthermore, cancer cells exhibit a high level of plasticity, based on the ability to dynamically switch from one cell type to another, especially in response to pharmacological treatments, triggering resistance mechanisms [106]. Thus, it is reasonable to assume that the different cancer cells or subclones constituting the tumor mass may show distinct sensitivity to SCD1 inhibitors, responding by activating/suppressing divergent molecular pathways. Based on these assumptions, such subclones can modulate the autophagic process in different ways.

Given the biological importance of autophagy and lipid metabolism, in particular SCD1, during cancer evolution, it is of utmost importance to deepen our understanding of their mutual interaction. In this context, a crucial experiment should involve tumor cells subjected to three types of drug treatments, namely SCD1 inhibitors, autophagy inhibitors and their combination, compared with untreated cells. Subsequently, the cells should be analyzed by single-cell RNA sequencing (scRNA-seq), as a valuable approach to unravel this controversial biological question. Indeed, by profiling single cells from a diversified population, scRNA-seq presents great advantages over traditional sequencing methods in dissecting heterogeneity, which is not detectable in bulk analyses and exploring rare cell types. In this specific case, this will allow us to define the tumor subpopulations fairly precisely, even the less represented ones, as well as to explore their evolution following pharmacological treatments. This approach may allow to understand if and how SCD1 and autophagy are related to each other, and potentially also to outline a shared molecular pathway.

This approach could also be adopted for in vivo studies. Patient-derived xenograft (PDX) models, in which tumor cells from a patient are implanted into immunodeficient mice, can be exposed to the drugs mentioned above (SCD1 inhibitors, autophagy inhibitors and their combination), and their tumor mass analyzed by a single cell transcriptomic analysis. Moreover, compared to in vitro studies, PDX models more faithfully represent the complexity of the human disease, including therapeutic responses to anti-cancer treatments.

A useful approach, complementary to scRNA-seq, is mass cytometry, a next-generation flow cytometry technique based on the use of antibodies conjugated to metal isotopes. This system allows the detection of more than 40 unique parameters, enabling the monitoring of many processes simultaneously and reveal co-regulation and crosstalk between cellular programs. Compared to scRNA-seq, mass cytometry is capable of measuring the post-translational modifications of the proteins, including phosphorylation, as well as identify different isoforms [107]. This method, integrated with the previously described scRNA-seq, can help address the intricate molecular signaling pathways linking SCD1 and autophagy, including stemness, inflammation, and ferroptosis.

Finally, for a comprehensive understanding of the topic, these approaches could be extended to cancer cells from different tissues, to highlight a possible tissue-specific differential signature.

## Targeting SCD1 and autophagy: clinical implications

SCD1 represents a promising target for new anti-tumor therapies. Several SCD1 inhibitors, including A939572, CAY10566, MF-438 and CVT-11127, have been tested as anticancer agents, both in vivo and in vitro*.* These drugs suppress proliferation and induce apoptosis in a number of cancer cell types, including kidneys, endometrium, liver, colon, breast and lung [61, 108–114]. Unfortunately, many of these efforts have remained at a pre-clinical level, failing to be translated to clinical trials. This is due, at least in part, to mechanism-based adverse events. Indeed, since the activity of SCD1 is critical for the production of sebum by the sebaceous glands, its inhibition leads to the atrophy of sebocytes, consequently causing eye dryness, hair loss and skin dryness [115, 116]. However, new SCD1 inhibitors administrable as “pro-drugs”, have recently been developed [117, 118]. Since sebocytes, unlike other cell types, are unable to activate the prodrugs into “active drugs” (irreversible steroyl-CoA inhibitors), these inhibitors may offer the opportunity to inhibit SCD1 more specifically in tumor cells, overcoming the side effects.

The targeting of autophagy also holds promise as an anticancer treatment, especially when combined with other anticancer strategies. At the clinical level, chloroquine or hydroxychloroquine (HCQ) have been proposed as autophagy-targeting agents [119]. Nevertheless, limited efficacy and toxicity are hindering their investigation, raising the need to develop more potent and specific autophagy inhibitors [120]. Toxicity, coupled with the limited poor efficacy, justifies the search for a new generation of agents targeting autophagy, which is currently in development, including Lys05, a bisamioquinoline, and DQ661, a dimeric quinacrine [121, 122]. Lys05 was found to be approximately tenfold more potent than HCQ, due to its greater accumulation within lysosomes where it deacidifies them [121]. Regarding Lys05, a remarkable antitumor efficacy was noticed in melanoma and colorectal adenocarcinoma in in vivo experiments, even as a single agent [121]. DQ661 was also shown to deacidify the lysosomes more than traditional anti-autophagy drugs, such as chloroquine and HCQ. This drug acts by inhibiting PPT1, a glycoprotein important in the catabolism of lipid-modified protein during lysosomal degradation. The rapid accumulation of palmitoylated proteins that occurs upon the inhibition of PPT1 alters mTOR signaling and lysosomal catabolism. It substantially translates into the reduction of tumor growth in melanoma, pancreatic cancer and colorectal cancer in mouse models [122]. However, clinical trials with Lys05 and DQ661 are not yet underway.

In the studies that directly addressed the relationship between SCD1 and autophagy in cancer, combined treatments with SCD1 inhibitors and autophagy regulators, both activators and inhibitors, were proposed [79, 80]. However, as we have discussed in this review, the choice of the most effective combination is not intuitive, and should be tailored on the specific biological context i.e., type of tissue, driver mutations, tumor heterogeneity, lipid vs sugar metabolism. Hence, it is essential to achieve a deeper understanding of the complex interaction between SCD1 and autophagy in order to identify the appropriate molecular background where this combined pharmacological approach may rationally be applied.

## Conclusion

Increasing evidence has shown that tumor cells have an altered lipid metabolism, affecting the production of the basic components of membranes, the synthesis and degradation of lipids for energy balance as well as the availability of lipid species with signaling functions [123]. The enzyme SCD1, necessary for the conversion of endogenous and exogenous saturated fatty acids into monounsaturated fatty acids, has been found to be up-regulated in several types of cancer [64–68]. Many studies have reported a role for SCD1 in promoting tumor growth and metastasis, as well as in maintaining stem cell-like phenotype [60–62].

**Fig. 5:**
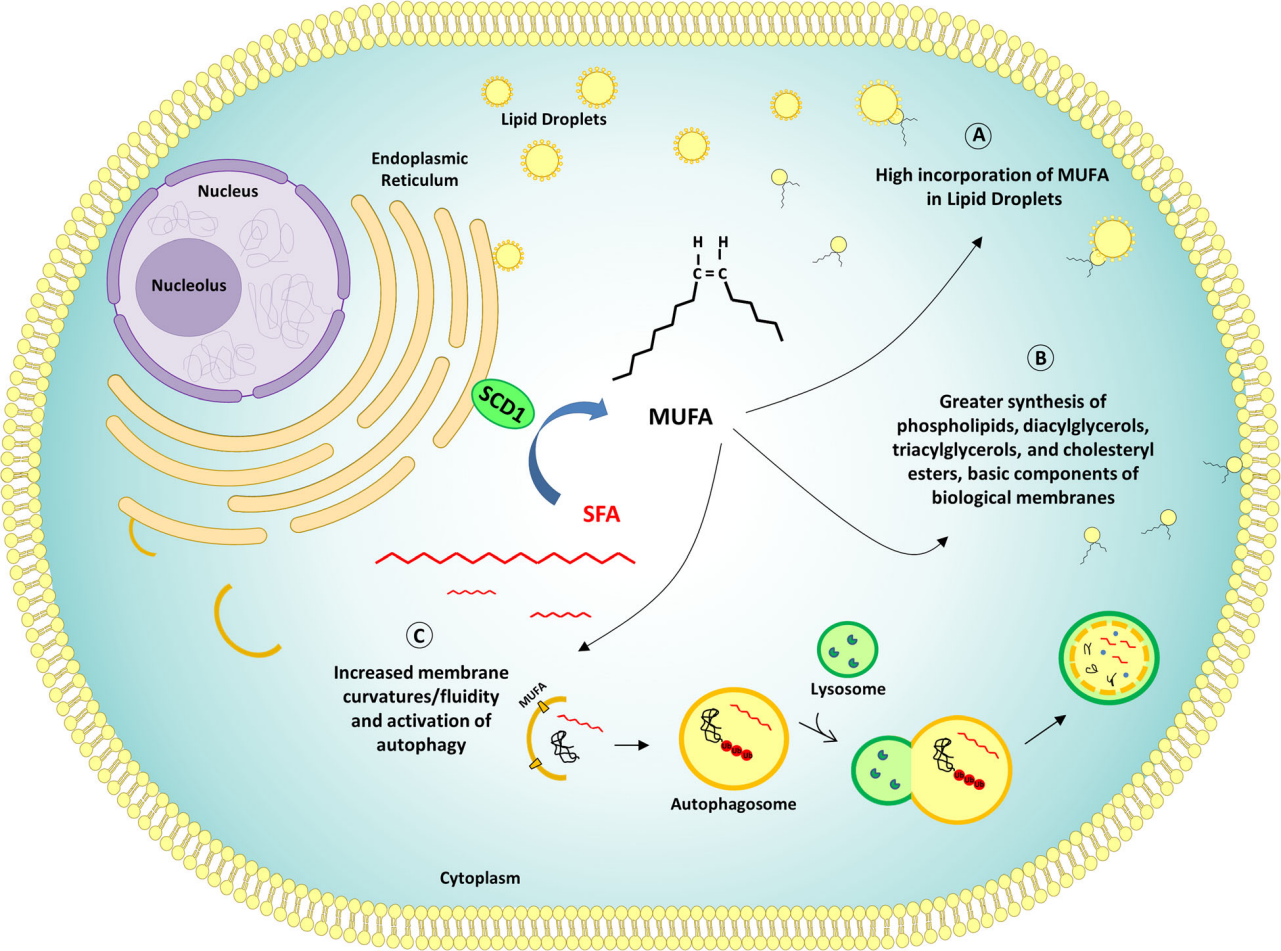
Role of SCD1 in MUFA synthesis and their contribution to lipid balance through autophagy regulation. SCD1 is an endoplasmic reticulum-bound enzyme that catalyzes the introduction of a double bond in the cis-9 position of saturated fatty acids (SFA), promoting the biosynthesis of monounsaturated fatty acids (MUFA) and a decreased SFA/MUFA ratio. The activity of SCD1 induces three main effects on lipid homeostasis of the cell, illustrated in the figure. **A** MUFA are more efficiently incorporated in lipid droplets compared to SFA; **B** MUFA are the substrates for the synthesis of various kinds of lipids, including phospholipids, diacylglycerols, triacylglycerols, and cholesteryl esters, basic components of biological membranes as well as cellular energy source and signalling molecules. **C** MUFA promote lipid bilayer fluidity and curvatures, facilitating the autophagosome formation on the ER and the activation of autophagy. In turn, in addition to removing damaged components, autophagy eliminates excess saturated fatty acids. These mechanisms counteract the cellular lipotoxicity and could be particularly important for the survival of cancer cells, especially Cancer Initiating Cells, which are characterized both by increased autophagy and the upregulation of SCD1

SCD1 is known to have an important role in regulating lipid bilayer fluidity and curvatures [124]. Furthermore, since MUFAs are incorporated at higher levels in lipid droplets as compared to SFA [125], SCD1 may be a protective factor against SFA-induced lipotoxicity. In this review, we highlight an additional role for SCD1, regarding the modulation of autophagy, both in normal and tumor cells. In Fig. 5, the main consequences of SCD1 activity and MUFA synthesis are illustrated.

The role of autophagy in cancer remains controversial: while it usually acts as a tumor suppressor allowing cells to remove damaged cellular contents, in other cases (often in later stages of tumor development) this mechanism helps cancer cells to survive under low oxygen and nutrient conditions, acting as a tumor promoter [25, 35]. Likewise, also the role of SCD1 in the regulation of autophagy in cancer is unclear, and further studies, aimed at clarifying the contribution of tumor heterogeneity, should be conducted. It is possible to hypothesize that in CICs or cancer cells undergoing EMT, characterized both by increased autophagy and the upregulation of SCD1 [38, 43, 70], the excess lipid content is kept under control, allowing them to resist stressful conditions. Considering that CICs and cells undergoing EMT are highly resistant to conventional cytotoxic therapies [39, 126], this hypothesis may have important clinical implications, providing the basis for the study of new combined anticancer strategies, involving both inhibitors of SCD1 and autophagy modulators.

## Data Availability

Not applicable.

## Abbreviations

3-NPA: 3-nitropropionic acid
AMPK: AMP-activated protein kinase
ATG: Autophagy-related protein
Bif-1: Bax-interacting factor 1
CE: Cholesteryl ester
CIC: Cancer-initiating cell
DAG: Diacylglycerol
EMT: Epithelial-mesenchymal transition
FFA: Free fatty acid
HCC: Hepatocellular carcinoma
HCC: Human hepatocellular carcinoma
HCQ: Hydroxychloroquine
LC3: Microtubule-associated protein 1A/1B-light chain 3
MAG-DHA: Docosahexaenoic acid monoglyceride
mTOR: mammalian target of rapamycin
MUFA: Monounsaturated fatty acid
PC: Phosphatidylcholine
PDX: Patient-derived xenograft
PE: Phosphatidylethanolamine
PI: Phosphatidylinositol
PI3K: Phosphoinositide 3-kinase
PI3P: Phosphatidylinositol 3-phosphate
PL: Phospholipid
PLD1: Phospholipase D
SCD: Stearoyl-CoA desaturase
scRNA-seq: single-cell RNA sequencing
SFA: Saturated fatty acid
SKO: Skin-specific deficiency of SCD1
TAG: Triacylglycerol
ULK1: Unc-51 Like Autophagy Activating Kinase 1
UVRAG: UV radiation resistance-associated gene protein
